# Variations in food and drink advertising in UK monthly women's magazines according to season, magazine type and socio-economic profile of readers: a descriptive study of publications over 12 months

**DOI:** 10.1186/1471-2458-11-368

**Published:** 2011-05-23

**Authors:** Jean Adams, Emma Simpson, Martin White

**Affiliations:** 1 Institute of Health & Society, Newcastle University, Newcastle upon Tyne, NE2 4HH, UK

## Abstract

**Background:**

Overweight and obesity are recognised nationally and internationally as key public health challenges. Food and drink advertising is one of the array of factors that influence both diet and physical activity choices and, hence, body weight and obesity. Little previous work has focused on food and drink advertising in magazines. We studied food and drink advertising in a wide range of popular UK monthly women's magazines published over a full year. We explored differences in the prevalence of food and drink advertising and the type of food and drinks advertised according to season, magazine type and socio-economic profile of readers.

**Methods:**

All advertisements in all issues of 18 popular UK monthly women's magazines published over 12 months were identified. For each food or drink advertisement, branded food and drinks were noted and categorised into one of seven food groups. All analyses were at the level of the individual advertisement.

**Results:**

A total of 35 053 advertisements were identified; 1380 (3.9%) of these were for food or drink. The most common food group represented was 'food and drinks high in fat and/or sugar' (28.0% of food advertisements), the least common group was 'fruits & vegetables' (2.0% of food advertisements). Advertisements for alcohol accounted for 10.1% of all food advertisements. Food and drink advertisements were most common in summer, general interest magazines, and those with the most affluent readerships. There were some differences in the type of food and drink advertised across season, magazine type and socio-economic profile of readers.

**Conclusions:**

Food and drink advertisements represented only a small proportion of advertisements in UK women's monthly magazines. Food and drink advertisements in these magazines feature a high proportion of 'less healthy' foods. There were a number of differences across season, magazine type and according to the socio-economic profile of readers in the prevalence of food and drink advertisements. Fewer differences were seen in the type of food and drinks advertised.

## Background

Overweight and obesity are recognised nationally and internationally as key public health challenges [1,2]. Advertising is one of the array of environmental and social factors that influence both diet and physical activity choices and, hence, body weight and obesity [3]. The role of television food advertising in food choice and obesity, particularly amongst children, has received substantial research attention over recent years [4,5], but a variety of other forms of food advertising exist. Magazines are a common source of diet-related information [6,7] and advertising in print media can reinforce brand images and communicate more detailed information than television advertising [8]. In 2007 around £92.4 m was spent on food advertising in print media in the UK - representing 19.1% of all food advertising spend in the UK [9].

Most published research on food advertising in magazines focuses on US publications [6,10-12]. This work suggests that food advertisements vary according to the population groups that different magazines are read by. For instance, US magazines aimed at white women contained more advertisements for fruit and vegetables, while those aimed at African American women contained more advertisements for alcohol [11]. These differences may contribute to the difference in body weight between these two groups, with white women tending to have a lower risk of obesity than African American women [13]. Differences in food advertising according to the socio-economic profile of magazine readers were also seen in recent work from the UK [14]. Fruit and vegetables and alcoholic beverages were more commonly advertised in magazines with more affluent readerships, whilst convenience foods were more commonly advertised in magazines with less affluent readerships [14].

Previous work on magazine food advertising tends to be limited in terms of the number of publications or consecutive issues studied [6,7,10-12,14,15]. Thus, results may be unduly influenced by one particular publication, or the time of year studied. To overcome these shortcomings, we studied food advertising in a wide range of popular UK monthly women's magazines published over a full year. We explored differences in the prevalence of food and drink (collectively referred to as 'food' throughout) advertising and the type of foods advertised according to season, magazine type and socio-economic profile of readers.

## Methods

### Publications of interest

All UK monthly magazines listed in the National Readership Survey (NRS) [16] as a women's magazine with a mean readership of over 500,000/month during October 2006 - September 2007 were considered for inclusion. Six publications were excluded - four supermarket magazines not readily available outside the supermarkets they are published by (*Asda Magazine*, *Sainsbury's Magazine*, *Somerfield Magazine *and *Waitrose Food Illustrated*) and two magazines aimed at teenagers (*Bliss *and *Sugar*) that are covered by statutory and voluntary regulations on food advertising to children [17]. Annual subscriptions to the remaining 18 publications were purchased in spring 2008 (Table 1).

**Table 1:** UK monthly women's magazines included in study

Publication	Cover price of first issue (£)	N issues per year	First issue in sample	Magazine type	Monthly readership **(1000 s)**^**1**^	**Percentage of population who read magazine**^**1,2**^			**C2DE:ABC1 ratio**^**6**^	**Affluence tertile**^**7**^
								
						Overall^3^	ABC1^4^	C2DE^5^		
BBC Good Food	3.10	12	April 2008	Food and homes	1149	2.4	3.2	1.3	0.41	Most
Company	2.00	12	May 2008	Lifestyle and beauty	647	1.3	1.6	1.0	0.63	Least
Cosmopolitan	3.20	12	April 2008	Lifestyle and beauty	1756	3.6	4.6	2.4	0.52	Middle
Country Living	3.50	12	April 2008	Food and homes	798	1.6	2.2	0.9	0.41	Most
Elle	3.30	12	May 2008	Lifestyle and beauty	854	1.8	2.2	1.2	0.55	Middle
Glamour	2.00	12	April 2008	Lifestyle and beauty	1236	2.6	3.3	1.7	0.52	Middle
Good Housekeeping	3.30	12	May 2008	General interest	1589	3.3	4.5	1.8	0.40	Most
Hair	2.99	10	June 2008	Lifestyle and beauty	677	1.4	1.5	1.3	0.87	Least
Homes & Garden	3.40	12	May 2008	Food and homes	878	1.8	2.3	1.1	0.48	Middle
House & Garden	3.60	12	April 2008	Food and homes	792	1.6	2.0	1.1	0.55	Least
House Beautiful	3.10	11	May 2008	Food and homes	505	1.0	1.4	0.6	0.43	Most
Ideal Home	3.10	12	May 2008	Food and homes	1050	2.2	2.6	1.6	0.62	Least
Marie Claire	3.20	12	May 2008	Lifestyle and beauty	1217	2.5	3.3	1.6	0.48	Middle
Mother & Baby	2.60	12	May 2008	General interest	678	1.4	1.2	1.6	1.33	Least
Prima	2.50	12	May 2008	General interest	684	1.4	1.6	1.2	0.75	Least
Red	3.30	12	April 2008	Lifestyle and beauty	693	1.4	2.1	0.6	0.29	Most
Vogue	3.80	12	April 2008	Lifestyle and beauty	1230	2.5	3.3	1.5	0.45	Most
Woman & Home	3.40	12	May 2008	General interest	813	1.7	2.2	1.1	0.50	Middle

^1^Data obtained from National Readership Survey (NRS), October 2006 - September 2007, restricted to adults aged 15 years or older, see reference[16]. ^2^As calculated by NRS. ^3^All adults aged 15 years and older. ^4^Adults aged 15 years and older in non-manual social classes, as defined and calculated by NRS. ^5^Adults aged 15 years and older in manual social classes, as defined and calculated by NRS. ^6^Ratio of percentage of population in manual social classes (C2DE) who read the magazine:percentage of population in non-manual social classes (ABC1) who read the magazine. ^7^Tertiles of C2DE:ABC1 ratio; most = most affluent readership, lowest C2DE:ABC1 ratio; least = least affluent readership, highest C2DE:ABC1 ratio; middle = intermediate readership, intermediate C2DE:ABC1 ratio.

### Data abstraction

All stand alone advertisements (excluding product placement in editorial content, money off coupons, advertorials, fliers and supplements) were identified and the product advertised and size of each advertisement (in printed pages) recorded. The term 'food advertisements' was used to describe all advertisements for branded food and drink products, but excluding dietary supplements and baby food. All branded products shown in food advertisements were recorded.

### Categorisation of food advertisements

Food advertisements were categorised into one of seven food groups, based on the five groups in the Food Standards Agency's 'EatWell Plate' [18] plus alcohol, and other products not otherwise classifiable ('other foods'). When more than one branded product was shown in an advertisement, categorisation was based on the most prominently displayed product (i.e. the largest). Within each food group, foods were also categorised into sub-groups to give an indication of the main types of food represented by each food group. These sub-groups were defined post-hoc (see Additional file 1, Table S1).

### Socio-economic profile of readers

As previously [14], National Readership Survey data on the social class breakdown of adult readers was used to calculate a metric reflecting the socio-economic profile of readers of each magazine: the ratio of the mean percentage of individuals in social classes C2, D and E (manual, less affluent social classes) who read the magazine each month to the mean percentage of individuals in social classes A, B and C1 (non-manual, more affluent social classes) who read the magazine each month. A higher "C2DE:ABC1" ratio indicates that the magazine tends to be read more by individuals in less affluent social classes than those in more affluent social classes. Magazines were grouped into three affluence tertiles based on this ratio (Table 1).

### Season and magazine type

Four seasons were assigned based on the cover month of publication: spring (March, April and May), summer (June, July and August), autumn (September, October and November), and winter (December, January and February). Three magazine types were assigned: lifestyle and beauty; food and homes; and general interest (Table 1).

### Analysis

Differences in the distribution of food categories across seasons, magazine types and affluence tertiles were explored using chi-squared tests. All analyses was performed using SPSS 15.0 and, due to the large number of tests performed, a p-value of <0.01 was taken to indicate statistical significance.

## Results

The total sample included 18 publications, 213 issues, 49 289 pages and 35 053 advertisements. Of these, 1380 (3.9%) were food advertisements. Table 2 shows the number of publications, cover price, issues, pages, advertisements and food advertisements broken down by season, magazine type and affluence tertile. There was evidence that the percentage of pages that were advertisements, the percentage of advertisements that were for food, the percentage of pages that were food advertisements, and the percentage of advertising pages that were for food all varied across season, magazine type and affluence tertile (p < 0.001 in all cases). Food advertisements were most prevalent in summer, general interest magazines, and magazines in the most affluent tertile and least prevalent in spring and magazines in the least affluent tertile. There was a statistically significant correlation between cover price and C2DE:ABC1 ratio (*r *= -0.50, *p *< 0.05) indicating that magazines with greater proportions of more affluent readers tended to be more expensive.

**Table 2:** Advertisements in UK monthly women's magazines overall and by season, magazine type and relative affluence of readers

	N publications	N issues	N pages		N adverts	N (%) pages that were adverts	N (%) adverts that were for food	N (%) pages that were food adverts	% advertising pages that were for food
All	18	213	49 289		35 053	19 543 (39.6)	1380 (3.9)	1452 (2.9)	7.4

Season									
Spring	18	52	12 145		8626	4812 (39.6)	236 (2.7)	241 (2.0)	5.0
Summer	18	54	11 519		8454	4445 (38.6)	400 (4.7)	423 (3.7)	9.5
Autumn	18	54	14 269		10 039	6079 (42.6)	428 (4.3)	450 (3.2)	7.4
Winter	18	53	11 356		7934	4207 (37.0)	316 (4.0)	339 (3.0)	8.1
χ^2 ^(p-value); df = 3						89.6 (<0.001)	49.9 (<0.001)	62.7 (<0.001)	71.5 (<0.001)

Magazine type									
Lifestyle & beauty	8	94	25 473		16 088	11 039 (43.3)	562 (3.5)	593 (2.3)	5.4
Food & homes	6	71	14 160		13 577	5202 (36.7)	362 (2.7)	370 (2.6)	7.1
General interest	4	48	9656		5388	3303 (34.2)	456 (8.5)	490 (5.1)	14.8
χ^2 ^(p-value); df = 2						314.4 (<0.001)	358.2 (<0.001)	192.4 (<0.001)	331.9 (<0.001)

Affluence tertile									
Least	6	71	12 882		9231	4256 (33.0)	255 (2.8)	259 (2.0)	6.1
Middle	6	72	20 057		12 227	8410 (41.9)	496 (4.1)	517 (2.6)	6.1
Most	6	70	16 350		13 595	6876 (42.1)	629 (4.6)	676 (4.1)	9.8
χ^2 ^(p-value); df = 2						318.5 (<0.001)	51.2 (<0.001)	129.7 (<0.001)	88.9 (<0.001)

Adverts = advertisements; df = degrees of freedom

Table 3 shows the distribution of food advertisements across the seven food groups, both overall and by season, magazine type and affluence tertile. In 261 (18.9%) food advertisements, more than one branded food product was shown. However, in all these cases all branded foods were in the same food group. The most common food group represented was 'food and drinks high in fat and/or sugar' (28.0% of food advertisements); the least common category was 'fruits & vegetables' (2.0%). There were significant seasonal variations in the proportion of food advertisements in the 'alcohol' and 'bread, rice, potatoes, pasta' groups. Advertisements for 'bread, rice, potatoes, pasta' were all most frequent in spring and least common in summer. Advertisements for 'alcohol' were most common in winter and least common in spring.

**Table 3:** Categories of branded foods advertised in UK monthly women's magazines overall and by season, magazine type and relative affluence of readers

	Alcohol	Bread, rice potatoes, pasta	Food & drinks high in fat and/or sugar	Fruits & vegetables	Meat, fish, eggs, beans	Milk & dairy foods	Other foods
All, n(%)	139 (10.1)	264 (19.3)	386 (28.0)	28 (2.0)	57 (4.1)	218 (15.8)	288 (20.9)

Season							
Spring, n(%)	9 (3.8)	64 (27.1)	53 (22.5)	6 (2.5)	18 (7.6)	36 (15.3)	50 (21.2)
Summer, n(%)	45 (11.3)	60 (15.0)	135 (33.8)	5 (1.5)	10 (2.5)	67 (16.8)	77 (19.3)
Autumn, n(%)	40 (9.4)	90 (21.0)	118 (27.6)	8 (1.9)	16 (3.7)	70 (16.4)	86 (20.1)
Winter, n(%)	45 (14.2)	50 (15.8)	80 (25.3)	8 (2.5)	13 (4.1)	45 (14.2)	75 (23.7)
χ^2 ^(p-value), df = 3	17.1 (0.001)	17.4 (0.001)	11.3 (0.010)	1.3 (0.721)	10.1 (0.017)	1.0 (0.801)	2.4 (0.498)

Magazine type							
Lifestyle & beauty, n(%)	114 (20.3)	69 (12.3)	165 (29.4)	5 (0.9)	15 (2.7)	91 (6.2)	103 (18.3)
Food & homes, n(%)	21 (5.8)	95 (26.2)	97 (26.8)	9 (2.5)	16 (4.4)	45 (12.4)	79 (21.8)
General interest, n(%)	4 (0.9)	100 (21.9)	124 (27.2)	14 (3.1)	26 (5.7)	82 (18.0)	106 (23.3)
χ^2 ^(p-value), df = 2	114.6 (<0.001)	31.2 (<0.001)	0.9 (0.630)	6.5 (0.038)	6.0 (0.051)	4.8 (0.091)	4.0 (0.138)

Affluence tertile							
Least, n(%)	7 (2.8)	57 (22.4)	81 (31.8)	5 (2.0)	8 (3.1)	36 (14.1)	61 (23.9)
Middle, n(%)	87 (17.5)	77 (15.5)	145 (29.2)	5 (1.0)	11 (2.2)	74 (14.9)	97 (19.6)
Most, n(%)	45 (7.2)	130 (20.7)	160 (25.4)	18 (2.9)	38 (6.0)	108 (17.2)	130 (20.7)
χ^2 ^(p-value), df = 2	51.6 (<0.001)	6.8 (0.033)	4.2 (0.121)	4.8 (0.091)	11.0 (0.004)	1.7 (0.423)	2.0 (0.373)

df = degrees of freedom

There were also significant variations in the proportion of food advertisements in the 'alcohol' and 'bread, rice, potatoes, pasta' food groups across magazine types. Advertisements for 'alcohol' were most common in lifestyle and beauty magazines and least common in general interest magazines. Advertisements for 'bread, rice, potatoes, pasta' were most common in food & homes magazines and least common in lifestyle and beauty magazines.

There were statistically significant variations across socio-economic tertiles in the proportion of food advertisements in the 'alcohol' and 'meat, fish, eggs, beans' categories. 'Alcohol' was most common in the middle tertile of magazines and least common in the least affluent tertile. Advertisements for 'meat, fish, eggs, beans' were most common in the most affluent tertile of magazines and least common in the middle tertile.

## Discussion

### Summary of findings

This is the first comprehensive study of the extent and nature of food advertisements in UK monthly women's magazines, covering a wide range of popular magazines published across a full year. Although advertising made up 40% of the pages of the magazines studied, food advertisements only accounted for 3% of magazine pages. As previously [6,7,10-12,14,15], 'less healthy' foods were prominent amongst food advertisements with 'food and drinks high in fat and/or sugar' accounting for more than a quarter of food advertisements. There were a number of variations in the prevalence of food advertisements according to season, magazine type and socio-economic profile of readers. Differences in the food categories that advertised foods fell into were less common. Advertisements for alcohol were not particularly common and accounted for only 10.1% of food advertisements.

### Strengths and weaknesses of methods

We included a much wider range of publications and issues than previously [6,7,11,15]. This allowed us to explore magazine food advertising across a wide variety of publications and to study differences in food advertising according to season and magazine type.

Our primary unit of analysis was the individual advertisement. This assumes that all advertisements have the same impact. However, food advertisements vary substantially and there are likely to be differences in the power of different advertisements to influence dietary choices - although it is difficult to see how this could be taken into account in this sort of study.

We only included branded products in our analysis and did not collect any information on non-branded food products shown in advertisements or elsewhere in magazines. Thus any incidental products shown in food advertisements (e.g. milk in an advertisements for a branded breakfast cereal) were excluded. These incidental products may have important influences on consumers', but to date this area has received little research attention. One study on television advertisements found that when non-branded food products were shown in advertisements they tended to be 'more healthy' than the branded products being promoted - suggesting that 'less healthy' branded products may be being promoted to a 'more healthy' food context in order to give them the 'air of healthiness' [19]. Future work could explore differences and similarities in branded and non-branded foods shown both in advertisements and elsewhere in magazines.

We used the food groups in the UK Food Standard's Agency's 'Eatwell plate' [18], plus two extra groups, to categorise advertised foods. Whilst this approach grounds our analysis in current UK public health context, many of the seven food groups used are quite diverse (see Additional file 1, Table S1). For example, the two most prevalent sub-categories in the 'food and drinks high in fat and/or sugar' food group are oils, fats and spreads and chocolate - two rather different types of food, eaten in different quantities on different occasions.

Furthermore, where individual foods fall within the 'Eatwell plate' categories, and our extension of them, may not always be obvious on first glance. We used the detailed 'Eatwell plate' guidance to help us with categorisation and introduced the category 'other foods' for those foods that we found difficult to place in one of the existing 'Eatwell plate' groups. Whilst there may be some apparent overlap between 'Eatwell plate' categories (e.g. cheese is categorised as 'milk & diary' but could also be considered 'high in fat and/or sugar'), in practice the detailed guidance provided on the categories clarifies where individual foods should be placed.

Although we could have used a categorisation scheme with more groups, this would arguably have made our results harder to interpret. No food group categorisation is perfect and there is always a balance to be struck between detail and interpretability.

### Interpretation of results

The monthly magazines studied devoted a similar proportion of their pages to food advertising as was found in a previous study of weekly magazines (3% here versus 4% in weekly magazines) [14]. However, whilst 16% of advertising pages were devoted to food in the weekly magazines, only 7% of advertising pages were found to be food in the monthly magazines studied here. Data from UK television indicates that around 15% of advertisements are for food [20]. Thus, food advertisements make up a relatively small component of the monthly magazines studied, and appear to make a smaller contribution to overall advertisement in monthly magazines than in other media. This highlights how different advertising in different media is and that results are not necessarily generalisable across media.

As with previous studies of print advertising [6,7,10-12,14,15], we found that food advertising in popular UK monthly women's magazines were dominated by foods in the 'food & drinks high in fat and/or sugar' group. The Eatwell plate suggest that 'balanced diet' contains "plenty" of 'fruit and vegetables', and 'bread, rice, potatoes, pasta'; "some" 'milk and dairy' foods and 'meat, fish, eggs, beans'; and "just a little" 'food and drinks high in fat and/or sugar'. Whilst it is difficult to compare our results directly to these rather non-specific suggestions, it is clear that food advertisements appear to over-represent 'food and drinks high in fat and/or sugar' and under-represent foods in all other groups, when compared to this definition of a 'balanced diet'.

One possible reason for the high volume of advertisements for 'food and drinks high in fat and/or sugar' and the low volume of advertisements for 'fruit and vegetables' is differences in what sorts of foods tend to be processed, and branded. For example, fruit and vegetables are rarely products that are branded, and when they are, this generally occurs when they have been processed in some way - e.g. dried, juiced, frozen. In contrast, 'food and drinks high in fat and/or sugar' tend to be highly processed foods where the opportunity for innovation and diversity, and hence branding, is ripe.

Some of the variations in food advertising seen across season may reflect social and cultural events associated with those seasons. For example, advertisements for alcohol were most prevalent in winter, presumably reflecting the impact of Christmas and New Year festivities when alcohol consumption tends to peak [21]. It is difficult to speculate whether there is a causal relationship operating here and, if so, in what direction. For example, it is possible both that increases in alcohol advertising around Christmas and New Year lead to increased consumption, or that advertisers merely reflect increased consumption at these times of year in their marketing. The reality is likely to be a mixture of both, with food and alcohol companies attempting both to increase their market share during times of peak consumption, and contributing to increased consumption through their marketing.

Similarly, differences in food advertising across magazine types may reflect the different readerships of different magazines. The only statistically significant differences in food advertisements between magazine types were in relation to the proportion of advertisements in the 'alcohol' and 'bread, rice, potatoes, pasta' categories. Alcohol accounted for more than 20% of food advertisements in 'lifestyle and beauty' magazines but was almost absent from other magazines. This may reflect the younger age of readers of 'lifestyle and beauty' magazines [16]. Again, it is likely both that marketers reflect the lifestyles of the readers in the magazines they target and that advertisements contribute to lifestyle differences between different demographic groups.

Differences in food advertising according to the socio-economic balance of readers of different magazines were not as marked as has been previously reported [14]. In particular, the clear trends for less healthy foods to be advertised more to less affluent readers and alcohol to be advertised more to more affluent readers, found in UK weekly magazines, were not seen here. Although, there was a non-significant trend for advertisements for 'food and drinks high in fat and/or sugar' to be more prevalent in magazines with less affluent readerships. This may be because of the much more affluent readership of monthly, compared to weekly, magazines. The C2DE:ABC1 ratio was <1 in all but one of the monthly magazines studied here and ≤0.5 in 50% of them. In contrast, it was ≥1 in 80% of weekly magazines studied [14]. It is possible that differences in food advertising according to the socio-economic profile of readers are not seen at the extremes of the socio-economic distribution that monthly magazines appear to represent. The clear association between our marker of the socio-economic balance of readers (C2DE:ABC1 ratio) and cover price indicates that this is likely to be an accurate marker of socio-economic position.

### Implications for policy and research

Although substantial work has now documented the extent of food advertising in different spheres [e.g. [4,5,14,22-24]], less work has focused on how food advertising is perceived and understood by both child and adult consumers. Further work is required to understand, for example, how different people respond to food advertisements both in print and elsewhere, and whether particular marketing techniques are particularly harmful in terms of effects on dietary preferences and consumption.

The advertising landscape and media is continually evolving. For example, there has been a decline in readership of many magazines over recent years. Comparison of the most recent readership figures from 2010 with those in 2007 (used to guide the research described here) indicate an average decline of around 10% across publications included in this research [16]. It is also possible that the recent introduction of regulations on television food advertising to children in the UK [25] has led to knock-on changes in food advertising in other media. Other recent developments include the rise of on-line and other non-traditional advertising, and new UK regulations allowing product placement on television made in the UK [26]. Policy makers and researchers need to be aware of the need to keep reappraising their strategies and knowledge in the light of industry developments.

Substantial research has confirmed that food advertising across a variety of different mediums tends to focus on 'less healthy' food [4,5,27]. The current work contributes to this evidence by highlighting the complexity of food advertising in relation to target audience and time of year. There is growing consensus that food promotion directed at children has a negative impact on food preferences and choice [4,5], and this has led to regulation of television food advertising to children in a number of contexts [28]. There is now also emerging evidence that adults are also influenced by food advertisements [29]. Although food advertisements were relatively rare in the magazines studied, by regulating food advertising in some, but not all, media, the UK government is sending mixed messages about its desire to tackle food marketing. A more consistent approach across all media may be warranted and it may now be time to consider regulation of all food advertising - not just that targeted at children, and not just that on television.

## Conclusions

Food and drink advertisements represented only a small proportion of advertisements in UK women's monthly magazines. Food and drink advertisements in these magazines feature a high proportion of 'less healthy' foods. There were a number of differences across season, magazine type and according to the socio-economic profile of readers in the prevalence of food and drink advertisements. Fewer differences were seen in the type of food and drinks advertised.

## Competing interests

The authors declare that they have no competing interests.

## Authors' contributions

JA & MW conceived the idea for this work. ES collected the data. JA & ES analysed the data. JA & ES drafted the manuscript. All authors reviewed and provided critical comments on previous versions of the manuscript. All authors read and approved the final manuscript.

## Pre-publication history

The pre-publication history for this paper can be accessed here:

http://www.biomedcentral.com/1471-2458/11/368/prepub

## Supplementary Material

Additional file 1 **Table S1 - proportions of food sub-categories within categories of branded foods advertised in UK monthly women's magazines**. Click here for file
