# Supplementary material for: Variations in food and drink advertising in UK monthly women's magazines according to season, magazine type and socio-economic profile of readers: a descriptive study of publications over 12 months
Source: BMC Public Health. 2011 May 23;11:368. doi: 10.1186/1471-2458-11-368 (PMC3121635; doi:10.1186/1471-2458-11-368)
Supplement: Additional file 1 — Table S1 - proportions of food sub-categories within categories of branded foods advertised in UK monthly women's magazines. [file 1471-2458-11-368-S1.DOC]

| Bread, rice, potatoes, pasta (n = 264) | | | Food & drinks high in fat and/or sugar (n = 386) | | | Meat, fish, eggs, beans (n = 57) | | | Other foods (n = 288) | |
| --- | --- | --- | --- | --- | --- | --- | --- | --- | --- | --- |
| Sub-category | N (%) | | Sub-category | N (%) | | Sub-category | N (%) | | Sub-category | N (%) |
| Breakfast cereal | 158 (59.8) | | Oil, fat, spreads | 113 (29.3) | | Lamb | 21 (36.8) | | Sauces | 85 (29.5) |
| Bread | 42 (15.9) | | Chocolate | 87 (22.5) | | Mycoprotein | 10 (17.5) | | Sweetener | 41 (14.2) |
| Crackers | 19 (7.2) | | Soft drinks | 56 (14.5) | | Beef | 9 (15.8) | | Stock | 36 (12.5) |
| Rice | 18 (6.8) | | Ice cream | 45 (11.7) | | Poultry | 7 (12.3) | | Water | 34 (11.8) |
| Potatoes | 16 (6.1) | | Conserves | 21 (5.4) | | Nuts | 5 (8.8) | | Instant coffee | 25 (8.7) |
| Cereal bars | 11 (4.2) | | Salty snacks | 20 (5.2) | | Pork | 3 (5.3) | | Other drinks (sugar free) | 22 (7.6) |
|  |  | | Biscuits | 18 (4.7) | | Meat | 2 (3.5) | | Other condiments (mustard) | 18 (6.3) |
|  |  | | Sweets | 10 (2.6) | | Pulses | 1 (1.8) | | Tea | 12 (4.2) |
|  |  | | Hot chocolate mix | 9 (2.3) | | Lamb & beef | 1 (1.8) | | Herbs & spices | 11 (3.8) |
|  |  | | Milkshake mix | 5 (1.3) | |  |  | |  |  |
|  |  | | Deserts | 2 (0.5) | |  |  | |  |  |
| Milk & dairy (n = 218) | | | Alcohol (n = 139) | | | Fruit & vegetables (n = 28) | | |  |  |
| Sub-category | | N (%) | Sub-category | | N (%) | Sub-category | | N (%) |  |  |
| Yoghurt, fromage frais | | 158 (72.5) | Spirits | | 66 (47.5) | Vegetables | | 9 (32.1) |  |  |
| Cheese | | 43 (14.9) | Wine | | 52 (37.4) | Fruit juice | | 9 (32.1) |  |  |
| Milk | | 17 (4.9) | Champagne | | 13 (9.4) | Dried fruit | | 8 (28.6) |  |  |
|  | |  | Beer, lager, cider | | 8 (5.8) | Fresh fruit | | 2 (7.1) |  |  |
